# The Proportion of Recent Thymic Emigrant Lymphocytes in Breastfed and Formula Fed Term Neonates

**DOI:** 10.3390/nu15041028

**Published:** 2023-02-18

**Authors:** Marco Lorenzini, Gergely Toldi

**Affiliations:** 1 School of Paediatrics, Thames Valley Deanery, Oxford OX4 2GX, UK; 2 Liggins Institute, University of Auckland, Auckland 1023, New Zealand; 3 Institute of Immunology and Immunotherapy, University of Birmingham, Birmingham B15 2TT, UK

**Keywords:** breastmilk, tolerance, neonate, newborn, regulatory T cells, naïve cells

## Abstract

Recent thymic emigrants (RTEs) represent a distinct T cell subset characterized by a tolerance-prone status. We have recently demonstrated that the proportion of regulatory T cells (Tregs) is nearly two-fold higher in exclusively breastfed compared with exclusively formula-fed neonates. However, it has been unknown whether the type of milk is also associated with the proportion of the RTE cell compartment. Cord blood (CB) and, at three weeks of age, peripheral venous blood samples were collected from 19 healthy-term neonates. A maternal blood sample was also taken. The proportion of RTEs, naïve CD4 cells, naïve RTEs, and Tregs was analyzed by flow cytometry in blood samples. RTE cell proportions were comparable between CB and 3 weeks. At both time points, there was no difference in the proportion of naïve CD4 cells, RTE CD4 cells, and naïve RTE CD4 cells between the feeding groups. The fold change of RTE cells between birth and three weeks of life was highest in mixed-fed babies. Since RTE counts were comparable across the feeding groups at birth, this most likely reflects a postnatal upregulation, to which the dual antigenic exposure to both non-inherited maternal antigens via breastmilk, as well as to other environmental antigens in formula milk, may contribute.

## 1. Introduction

Postnatal adaptation necessitates that the immune system undergoes a rapid shift from relative quiescent cell division to active postnatal protection against pathogens while simultaneously maintaining tolerance to commensals. Therefore, besides pro-inflammatory lymphocyte subsets, tolerogenic immune cell subtypes also play a major role in the successful postnatal adaptation of the immune system and evolving adaptive immune responses.

Recent thymic emigrants (RTEs) represent a distinct T cell subset undergoing post-thymic maturation. These cells, which have completed their development in the thymus and subsequently egressed from it, play a critical but still understudied role in connecting the thymic output to the recirculating T cell pool. They form a considerable proportion of the T cell population in neonates, infants, and young adults [1]. In comparison to mature naïve T cells, RTE are functionally different, as the former show lower proliferation, cytokine production, and expression of early activation markers (including CD25) upon antigen encounter [2].

Cunningham et al. described RTEs as a group of cells uniquely adapted to the environment they encounter upon thymic egress. They have enhanced tissue invasiveness, and their trafficking maximizes exposure to extrathymic self or commensal-derived antigens; thus, their tolerance-prone status reduces the risk of autoimmunity. As such, RTEs are not flawed mature T cells but are adapted to fill an underpopulated T cell compartment while maintaining self-tolerance but also being capable of mounting a robust immune response. They are also characterized by an enhanced ability to acquire a regulatory T cell (Treg) phenotype. Additionally, deletion tolerance and anergic propension also contribute to their tolerogenic profile [3].

RTEs are clinically important as their biology is fundamental to predicting the responses to the immunological challenge of neonates, aging individuals, and those recovering from lymphoablation. In addition, RTEs may have a role in several disease states in adults, including ulcerative colitis, chronic myeloid leukemia, and autoimmune thyroid disease [1]. However, their role in inflammatory complications affecting preterm and term neonates, or the increased susceptibility to infections in the neonatal period, is not well described. A recent prospective study of a birth cohort of preterm and term neonates (ranging from 23 to 42 weeks of gestation) demonstrated that mid to late human gestation marks a critical period of antenatal lymphocyte development, during which accumulation of CD31+ IL-8+ RTEs occurs. Prematurity presumably perturbs this critical step of maturation. Indeed, preterm neonates born at less than 29 weeks of gestation who failed to achieve an age-appropriate T cell phenotype by term-corrected gestational age, presenting with a decreased proportion of CD31+ IL8+ RTE cells in comparison with full-term infants, were at a 3.5-fold greater risk of respiratory morbidity in their first year of life after discharge from the neonatal unit [4].

Tregs are a subtype of mature T cells that play a crucial role in maintaining immunologic self-tolerance and negatively regulating inflammatory immune responses [5]. Reduced Treg numbers are associated with pathologies such as necrotizing enterocolitis and bronchopulmonary dysplasia in the preterm population [6,7]. We have recently demonstrated in a cohort of healthy, term neonates that the proportion of Tregs increases in the first three weeks of life and is nearly two-fold higher in exclusively breastfed neonates compared with exclusively formula-fed neonates. In addition, breastfed neonates show a specific and Treg-dependent reduction in proliferative T cell responses to non-inherited maternal antigens (NIMA) and have reduced inflammatory cytokine production [8]. These findings indicated that postnatal exposure of the neonate to maternal antigens through breastfeeding acts to drive the maturation of Tregs. However, it has been unknown whether the type of nutrition the newborn receives in the first weeks of life (i.e., breastmilk versus formula) is also associated with the proportion of the RTE cell compartment.

While RTEs primarily maintain a tolerogenic immunological profile, they also play an important role in initiating and balancing pro-inflammatory responses in the early postnatal weeks of life by the production of a potent pro-inflammatory cytokine, IL-8 [4,9]. They, therefore, have a crucial contribution to maintaining the immunological defense against pathogens in this vulnerable period of life while contributing to immunological tolerance of commensals and self-antigens.

The aim of this study was, therefore, to establish the differences in the proportion of RTE cells at three weeks of age in healthy, term neonates depending on the type of milk they receive. We hypothesized that similarly to Tregs the proportion of tolerogenic RTE cells is also increased in breastfed compared with formula-fed neonates. 

## 2. Materials and Methods

### 2.1. Participants

This was a retrospective analysis of a cohort of 19 healthy term neonates born by elective cesarean section who were part of a larger study focusing on the immunological effects of breastfeeding [8]. All neonates who had sufficient blood samples to perform the flow cytometry analysis described below from the larger study were included. Venous cord blood (CB) and, at three weeks of age, peripheral venous blood samples were collected from neonates. Maternal peripheral blood samples were also collected prior to the caesarean section. Nine neonates were exclusively breastfed (BF) throughout the study period, while five and five neonates were mixed-fed (MF) and exclusively formula-fed (FF), respectively. The formulas used were of various brands. All formulas were cow’s milk-based stage 1 term infant formulas. 

Exclusion criteria included multiple pregnancies, clinical risk factors for maternal sepsis (especially maternal fever, chorioamnionitis), Group B Streptococcus positivity in the current pregnancy, known genetic conditions of the fetus or the mother, and maternal HIV, TB, new-onset viral infection, hypertensive disorder, endocrine condition/diabetes, asthma, autoimmune conditions, and medication use aside from pregnancy supplements. The study was reviewed and approved by the East Midlands – Nottingham 2 NHS Research Ethics Committee (reference 16/EM/0379). Informed written consent was obtained to participate in the study from all women prior to delivery.

### 2.2. Immunophenotyping

Peripheral blood mononuclear cells (PBMCs) from blood samples were isolated and stored as described earlier [8]. Flow cytometry analysis was performed in batches. Cells were surface stained in MACS buffer with BV510-conjugated CD4, ECD-conjugated CD14/CD19/CD56, AF700-conjugated CD45RA, APC-Cy7-conjugated CD3, BV421-conjugated CD25, BV605-conjugated CD31, PE-conjugated PTK7, FITC-conjugated CCR7 (all from Biolegend, San Diego, CA, USA), and Live/Dead viability dye (red, 488 nm, Invitrogen, Carlsbad, CA, USA). After washing, cells were fixed with fixation/permeabilization solution (eBioscience, San Diego, CA, USA) for 30 min at room temperature in the dark. Cells were washed with permeabilization buffer (eBioscience), and AF647-conjugated FoxP3 (Biolegend) antibodies were added for 30 min in the dark. Cells were then washed and run on an LSRII flow cytometer equipped with blue, red, and violet lasers (BD Biosciences, San Jose, CA, USA). At least 50,000 cells were recorded per sample. Unstained and single-stained samples were used as compensation controls.

During the gating process, doublets were first excluded based on FSC-A and FSC-H characteristics. Lymphocytes were then identified based on FSC-A and SSC-A characteristics. Dead, as well as CD14+, CD19+, and CD56+ cells were excluded based on positivity in the ECD channel. Further gating was performed within CD3+ cells. Naïve CD4 cells were identified as CD4+ CD45RA+ CCR7+, RTE CD4 cells were identified as CD4+ CD31+ PTK7+, while regulatory T cells (Tregs) were identified as CD4+ CD25hi FoxP3+. Flow cytometry data were analyzed using FlowJo 10.8.1 software.

### 2.3. Statistical Analysis

Comparisons were made using the Friedman and Kruskal-Wallis tests as the distribution of data appeared to be non-normal according to the Shapiro-Wilk test. Correlation analysis was performed using Spearman’s correlations. *p* values < 0.05 were considered significant. Analysis was performed using GraphPad Prism 9 software.

## 3. Results

Characteristics of participating neonates and mothers are summarised in Table 1. Anthropometric and pregnancy data were comparable across the feeding groups.

**Figure 1:**
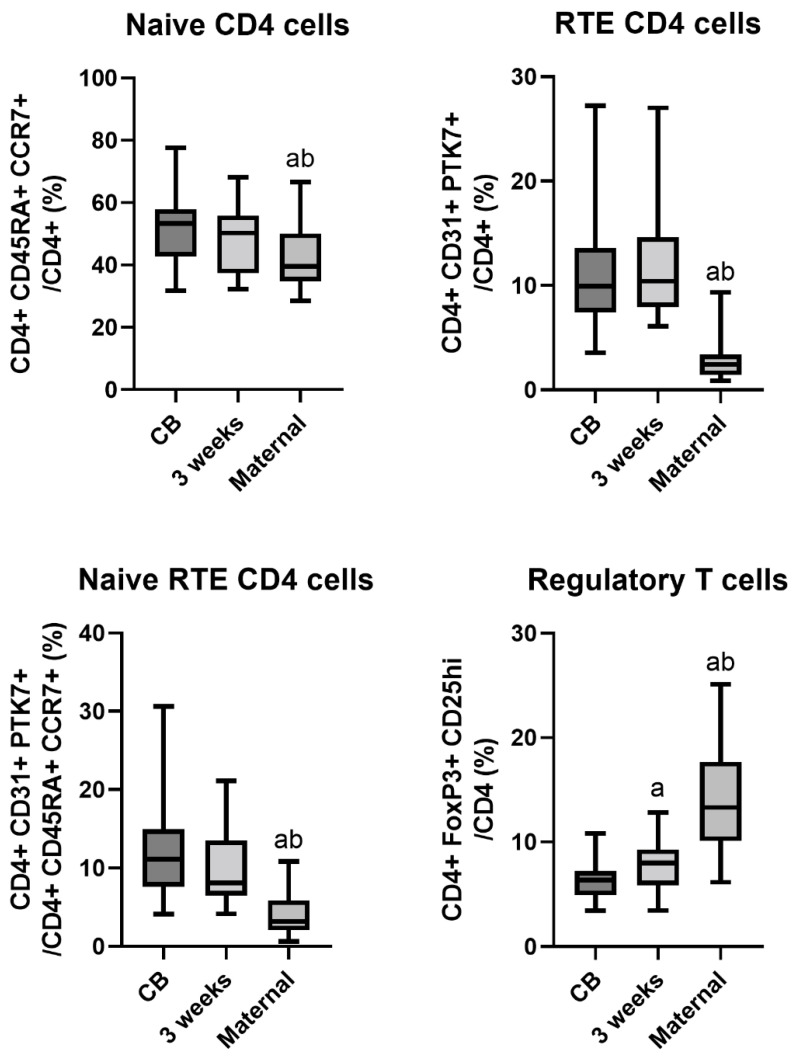
The proportion of investigated cell subsets in cord blood (CB), neonatal blood at three weeks of age, and maternal blood (*n* = 19 in each group). *p* < 0.05 vs. a—CB, b—three weeks. Line — median, box—quartiles, whiskers—range.

The proportions of naïve CD4 cells, RTE CD4 cells, and naïve RTE CD4 cells were higher in neonatal than maternal samples and were comparable between CB and three weeks. The proportion of Tregs was highest in maternal samples, as expected during pregnancy. An increase in the proportion of Tregs by three weeks of age was also noted in comparison to CB (from 6.4% to 8.0%), as reported earlier [8] (Figure 1).

**Figure 2:**
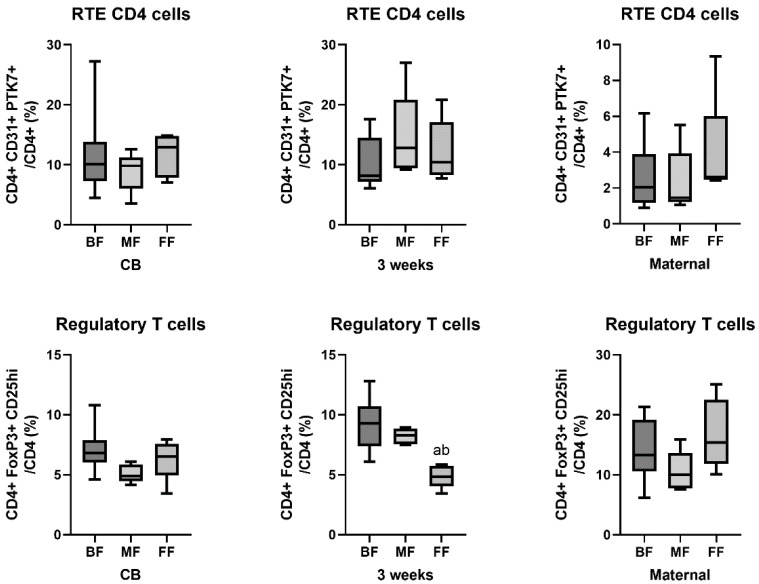
The proportion of recent thymic emigrant (RTE) and regulatory T cells in cord blood (CB) and at three weeks of age in neonates as well as in maternal blood before delivery according to the feeding method (BF—exclusively breastfed (*n* = 9), MF—mixed fed (*n* = 5), FF—exclusively formula fed (*n* = 5)). *p* < 0.05 vs. a—BF, b—MF. Line—median, box—quartiles, whiskers—range.

Next, we examined the differences in cell subset proportions between BF, MF, and FF babies. We also grouped and examined maternal blood samples according to the feeding method to rule out potential underlying differences due to maternal biology. In CB and at three weeks of age, as well as in maternal samples, there was no difference in the proportion of naïve CD4 cells, RTE CD4 cells, and naïve RTE CD4 cells between the three feeding groups (Figure 2).

While the proportion of Tregs was comparable between the three feeding groups in CB and maternal samples, by three weeks of age the frequency of Tregs was nearly twofold higher (9.3% vs. 4.9%) in BF compared with FF neonates, as previously reported [8]. The proportion of Tregs in samples from babies receiving MF was more comparable to that of BF babies than FF babies (Figure 2).

**Figure 3:**
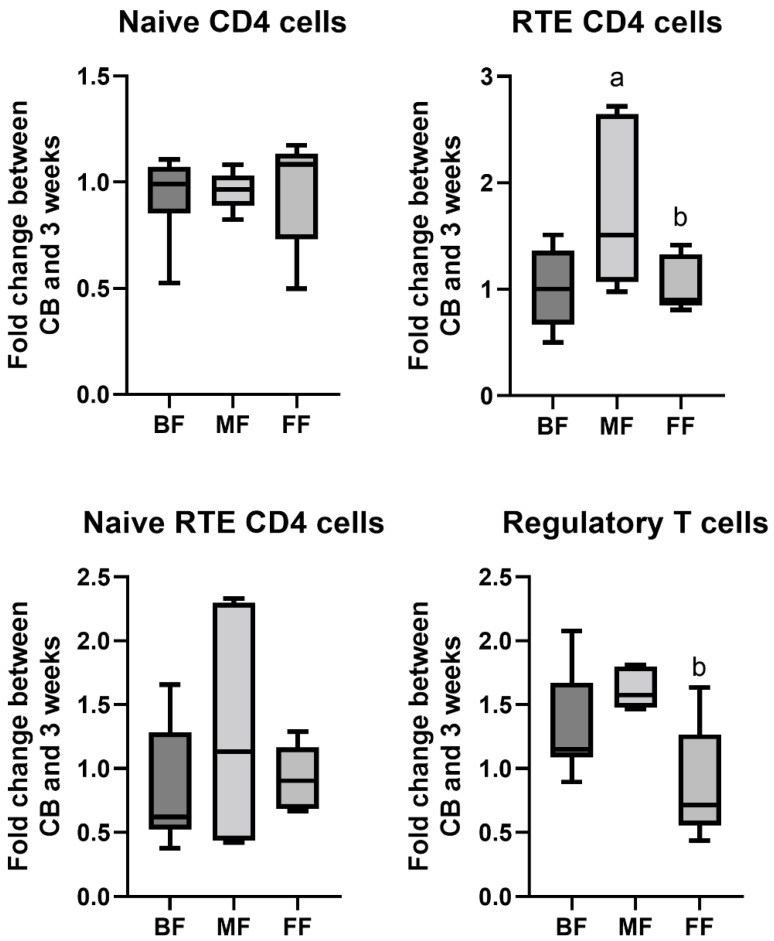
Fold change of the investigated cell subsets in neonates between birth and three weeks of age according to the feeding method (BF—exclusively breastfed (*n* = 9), MF—mixed fed (*n* = 5), FF—exclusively formula fed (*n* = 5)). *p* < 0.05 vs. a—BF, b—MF. Line—median, box—quartiles, whiskers—range.

We also compared the fold change of the examined cell subset proportions amongst the feeding groups, reflecting the rate of increase or decrease between birth and three weeks. The highest fold change in Tregs was noted within the MF group, closely followed by BF babies. Interestingly, in the case of RTE cells, the fold change was highest in MF compared to both BF and FF babies. No differences were noted in fold changes between the feeding groups in case of naïve and naïve RTE CD4 cells (Figure 3).

**Figure 4:**
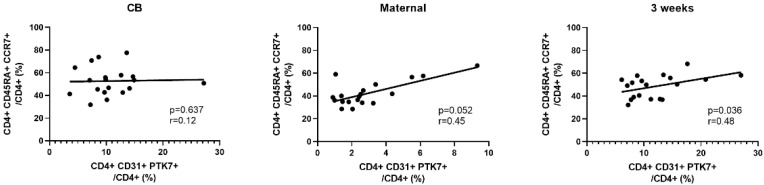
Spearman’s correlations of the proportion of recent thymic emigrants (RTE, horizontal axis) and naïve CD4 cells (vertical axis) in cord blood (CB), maternal blood, and neonatal blood at three weeks of age (*n* = 19 in each group).

Finally, we examined the correlation of the proportion of RTE cells with the other cell subsets. A positive correlation was noted between RTE and naïve cells as well as between RTE and naïve RTE cells at three weeks. Interestingly, in CB samples the correlation was only present between RTE and naïve RTE cells but not between RTE and naïve cells (Figure 4).

## 4. Discussion

We reported earlier that the proportion of Tregs, which have a functionally similar role in suppressing pro-inflammatory responses, is higher in breastfed babies compared to those who only receive formula after birth [8]. We, therefore, hypothesized that the proportion of RTEs might also differ depending on the type of feeding. While we demonstrated that the proportion of RTEs is indeed higher in the neonatal period (comparable at birth and at three weeks of age) compared to adult samples, no significant impact of the feeding type was detectable. While the proportion of Tregs increases from birth to three weeks, that of RTEs seems to be stable during this period. As expected, naïve CD4 cells are more prevalent in neonatal than in adult samples.

While the fold change in Tregs was highest in MF, closely followed by BF babies, the fold change in RTEs was outstandingly high in MF compared to BF or FF. This might indicate that for the expansion of this subset in early life, a dual antigenic exposure to both non-inherited maternal antigens (NIMA) via breastmilk as well as to other antigens, such as cow’s milk protein (CMP) antigens present in formula is necessary. Similarly to that of Tregs, the impact of the early expansion of RTEs is likely beneficial on long-term immune health, including the incidence of allergies and autoimmune disorders in later life, as they help achieve a more established immune tolerance towards commensals and other harmless environmental antigens.

RTEs are the youngest peripheral T cells susceptible to peripheral T cell tolerance mechanisms. We observed a lack of correlation between RTE and naïve CD4 cell proportion at birth in comparison to three weeks of life. This may indicate a temporary perturbance of or not yet established RTE function, where RTEs contribute to the balance of differentiation from a naïve to effector and memory phenotypes [10]. 

The clinical significance of the above findings is currently unknown. The long-term relevance of the postnatal upregulation of RTEs in mixed fed babies may seem to be questionable, as the proportion of this cell type ceases significantly by adulthood. However, recent findings do indicate an effect of RTEs on immune health in adult life, reporting reduced frequencies of detectable RTEs in ANA-positive patients. The observed decreased frequencies of RTEs in ANA-positive patients in this study may be due to premature differentiation into effector T cells and reflect a failure of tolerance mechanisms [11].

Limitations of our study include the low number of participants, especially within each feeding type subgroup. On the other hand, participants represent a unique cohort of healthy neonates without conditions that could have possibly influenced the results, sampled exclusively for research purposes both at birth and at three weeks of age. A further limitation of this observational study is the lack of mechanistic insight with regards to the presumed factors influencing the prevalence of RTEs in the early neonatal period. However, our results may form the basis of future studies investigating such mechanisms.

Overall, RTE cell counts appear to be stable in the first three weeks of life. However, MF babies seem to have the highest increasing fold change of RTEs in this period. Since RTE counts were comparable across the feeding groups at birth, this is more likely to reflect a postnatal upregulation, to which the dual antigenic exposure to both NIMA via breastmilk as well as to other environmental antigens, such as CMP present in formula milk likely contributes. This may have a long-term positive impact on evolving tolerance mechanisms lasting well beyond the neonatal period.

## Figures and Tables

**Table 1:** Anthropometric and pregnancy characteristics of participating neonates and mothers. Data are presented as median [range] or ratio.

	All Neonates *n* = 19	Breastfed *n* = 9	Mixed Fed *n* = 5	Formula Fed *n* = 5
Gestation (weeks)	39 [39–39]	39 [39–39]	39 [39–39]	39 [39–39]
Birth weight (g)	3495 [2960–4590]	3495 [2960–4100]	3440 [2980–4590]	3500 [3120–4500]
Weight at visit (g)	3720 [3160–4760]	3405 [3160–4440]	3970 [3370–5050]	3740 [3510–4760]
Age at visit (days)	21 [19–23]	21 [19–23]	21 [20–23]	21 [20–23]
Sex (male/female)	11/8	5/4	3/2	3/2
Maternal age (years)	31 [26–47]	31 [27–37]	31 [29–47]	32 [26–41]
Parity (primi/multi)	4/15	2/7	1/4	1/4

## Data Availability

The data presented in this study are available on request from the corresponding author. The data are not publicly available due to privacy restrictions.
